# Social Engagement, Depressive Symptoms, and Loneliness, and Longitudinal Cognitive Decline in a Population-Based Cohort of Older Adults

**DOI:** 10.1016/j.osep.2025.02.004

**Published:** 2025-03-28

**Authors:** Pankaja Desai, Ted K.S. Ng, Kristin R. Krueger, Robert S. Wilson, Denis A. Evans, Kumar B. Rajan

**Affiliations:** Rush Institute for Healthy Aging (PD, TKSN, KRK, RSW, DAE, KBR), Rush University Medical Center, Chicago, IL; and the Rush Alzheimer’s Disease Center (RSW), Rush University Medical Center, Chicago, IL.

**Keywords:** Cognitive function, Cohort study, Depression, Epidemiology, Loneliness

## Abstract

**Objective:**

The primary objective of this study is to examine the association between social engagement and cognitive decline among participants with and without depressive symptoms and/or loneliness.

**Methods:**

Study data is from the Chicago Health and Aging Project (CHAP), a population-based cohort study, located on the south side of Chicago, which consisted of interviews occurring every 3 years from 1993 to 2012. We conducted mixed effects regression analysis to examine the association between social engagement and global cognitive decline in CHAP participants. Models adjusted for and were stratified by depressive symptoms status and loneliness status.

**Results:**

The study sample includes 10,572 participants (63% black and 61% female). A total of 2,481 participants experienced more depressive symptoms, and 1,751 participants were lonely. A higher frequency of social engagement was associated with a slower rate of cognitive decline in individuals with and without depressive symptoms and/or loneliness. Participants who were lonely had approximately a 17% (β = 0.009/β = −0.054) slower rate of cognitive decline, and participants who were not lonely had a slower rate of about 12% (β = 0.006/β = −0.051), for a one-unit increase in social engagement. For each unit of increase in social engagement, participants with greater depressive symptomology had a slower rate of cognitive decline of approximately 14% (β = 0.009/β= −0.063), and participants with no to few depressive symptoms had a slower rate of decline of about 12% (β = 0.006/β = −0.049).

**Discussion:**

Social engagement slows cognitive decline in individuals with loneliness and/or depression. It is essential to build strategies for adhering to social activities into interventions that seek to minimize risk of cognitive impairment.

## BACKGROUND

Social engagement is the foundation for developing social connections and taking part in one’s community.^1^ Loneliness is defined as a *subjective feeling of being alone*.^2^ Roughly 43% of older adults in the United States are lonely,^3^ and about 8% of adults reported having at least one depressive episode in 2021.^4^ Approximately 6.9 million adults, 65 years of age and older, have Alzheimer’s disease in the United States.^5^ Depression, and dementia are both highly prevalent in older adults.^6^ Our previous work showed that black older adult participants indicated 60% greater depressive symptomology than white older adult participants.^7^ It is important to note that cultural aspects provide context for socialization in older adults, including viewpoints of how one lives or of getting older.^8^

The central theme of this study is whether or not social engagement influences cognitive trajectories differently over time in individuals with or without depressive symptomology and/or loneliness. A review conducted to evaluate the relationship between social engagement, feelings of loneliness, and dementia risk reports inverse relationships between increased social engagement, social support, and social networks and greater risk of dementia.^9^ Social networks, activities, support, and relationships benefit global cognition.^10^ Interventions that improve loneliness in older adults residing in the community seem to include treatments that are group-oriented, online and may perhaps include exercises which are group-based.^11^ Greater social engagement in older adults is associated with improved wellbeing and protects against dementia.^9,12,13^ It is also important to note that there may be differences in how social engagement may benefit cognitive functioning, depending on whether or not individuals experience depressive symptoms.^14^ For instance, major depressive disorder may hinder social cognitive ability, and individuals with this disorder may experience challenges with social interaction because of how they comprehend certain stimuli.^14^

The Longitudinal Aging Study in India conducted a cross-sectional analysis and found that depressive symptomology confounded the association between social engagement and cognitive function.^15^ Findings support the maintenance of social connectivity to benefit both cognitive function and mental health among older adults.^15^ One framework indicates that social networks are behavioral, functioning through pathways of social engagement and social support, which influence depressive symptoms, stress responses that are physiological, and lifestyle behaviors, which increase the risk of conditions resulting in cognitive decline.^16,17^ We follow a component of this framework by testing the effect modification of depressive symptoms or loneliness in the association between social engagement and global cognitive decline in older adults. We hypothesize that increased social engagement slows the rate of cognitive decline in individuals with fewer depressive symptoms or who are not lonely. It is also important to understand the relationship between social engagement and cognitive decline in older adults who specifically experience depression or loneliness to inform the development of interventions tailored for individuals experiencing these symptoms who may be in more need of such interventions which benefit cognitive health.

It is important to note that depression, loneliness, and symptoms of Alzheimer’s disease are interrelated.^18^ For instance, cognitive difficulties can occur in depression and Alzheimer’s disease.^18^ Limited work has been conducted to understand the relationships between these three conditions.^18^ Our study informs this gap in knowledge by examining the association between social engagement and cognitive decline in individuals with and without depression and/or loneliness.^18^ Studies have evaluated the risk of depression in Alzheimer’s disease.^19,20^ Depression in middle age is associated with dementia.^19^ Depression is also a modifiable risk factor for mild cognitive impairment and dementia.^20^ Studies have also examined the relationships between loneliness and cognitive function.^21^ Among people who are screened for dementia, loneliness is associated with adverse cognitive performance across several domains, suggesting that we need to improve our understanding of the role of loneliness in the pathway to AD.^21^ This study explores the role of depressive symptoms and loneliness in the association between social engagement and cognitive decline over time in a cohort study of black and white participants to inform future interventions.

## METHODS

### Study Sample

The study sample is comprised of participants from the Chicago Health and Aging Project (CHAP), a population-based cohort study of older adults residing in the south side of Chicago. This analysis includes CHAP data collected every three years from 1993 to 2012 in four communities located on the south side of Chicago. Participants were recruited through a door-to-door census in the four communities of focus and had to be 65 years or older at the baseline interview.^22^ A total of 10,802 participants were recruited in CHAP. Participants included in this analysis (*N* = 10,572) completed the social engagement measure, the depression measure, and at least one cognitive assessment. The mean study follow-up was 8.8 years, with a maximum follow-up of 18.7 years.

### Measures

#### Social Engagement.

Items from the perceived social engagement measure are from the *Established Populations for Epidemiologic Studies of the Elderly Resource Data Book*.^23^ This measure is coded as follows: *1. Are you currently working at a job? (0 = No, 1 = Part-Time, 2 = Full-time); 2. How often do you go to religious services? 3. How often do you go to museums? (items 2 and 3: 0 = Once a year or less, 1 = Several times a month or several times a year, 2 = Every day or several times a week); 4. Do you participate in any activities or groups outside the house, such as a senior center, public service, etc.? (0 = No, 2 = Yes)*.^22–26^ Responses are totaled to obtain a score ranging from 0 to 8.^22–26^

#### Depression and Loneliness.

An adapted form of the Center for Epidemiologic Studies-Depression (CESD) scale was used to measure depressive symptomology.^27−29^ Participants were asked to respond (*0 = No and 1 = Yes*) to this question per item: *Have you felt this way much of the time during the past week?* The instrument includes the following items: 1) *I was happy 2) I enjoyed life 3) I felt sad 4) I felt lonely 5) I felt depressed 6) I could not get going 7) My sleep was restless 8) I felt like everything I did was an effort 9) I felt that people disliked me 10) People were unfriendly*.^27−29^ A score with a range of 0−10 was calculated by summing items. Participants with a score of 3−10 were defined as having more depressive symptoms, and participants with a score of 0−2 were defined as no to few depressive symptoms. This categorization was determined based on the frequency distribution of values with lower scores having greater frequencies than higher scores and our previous work in this study which also used this cut point.^30^ Loneliness was also examined separately using the item: *I felt lonely* and again, scored as *0 = No and 1 = Yes*.^27–29^ The adapted CES-D measure has been validated in adults greater than 60 years of age, with a sensitivity of 100%, specificity of 93%, and positive predictive value of 38%.^31^

#### Cognitive Functioning.

Global cognitive functioning was assessed utilizing the East Boston Memory Test—Immediate Recall and Delayed Recall for measuring episodic memory, the modified and oral version of the Symbol Digit Modalities Test for measuring perceptual speed, and the Mini-Mental State Examination (MMSE).^32–37^ The means and standard deviations of each measure at baseline were used to calculate Z-scores. The average of the z-scores across the measures was then taken to calculate the global cognitive function score.^32–37^

### Statistical Analysis

Analysis was conducted using SAS v. 9.4. Box plots were developed to determine the distribution of social engagement by loneliness and depressive symptom status. We also computed baseline descriptive statistics using means and standard deviations for continuous variables and percentages and frequencies for categorical characteristics. The Student’s t-Test was conducted to examine mean differences of descriptive characteristics by depression or loneliness status. The Pearson’s Chi-Squared Test was conducted to examine race and sex differences by depression or loneliness status. Linear mixed effects regression models were implemented to test the associations between the frequency of social engagement and global cognitive decline. We tested the interaction of social engagement and loneliness and the interaction of social engagement and depression status. *β* coefficients were unstandardized.

Stratified models for loneliness status (*1 = Yes and 2 = No*) and for depression status (<3: no to few symptoms, ≥3: more symptoms) were also implemented to examine associations between social engagement and global cognitive decline within loneliness and depressive symptomology categories. We conducted stratified models by loneliness and depression status to better understand the within-group associations between social engagement and global cognition. We evaluated within-group associations to determine if social engagement interventions should be tailored to more lonely or depressed individuals. All models adjusted for demographic characteristics, including age, sex, race, education in years, time in years, and their interactions with time to reduce bias in the association between social engagement and global cognition. All predictors in the models, excluding time, were measured at baseline, and their associations with the outcome of global cognitive function were examined. The interaction of each predictor with time examined associations with global cognitive decline. The global cognitive decline over ten years was plotted by loneliness status and separately by depression status for individuals at the 10th and 90th percentiles of social engagement.

## RESULTS

Table 1 shows the baseline characteristics of 10,572 study participants (63% black race and 61% female sex). Participants with more depressive symptoms were significantly older and had fewer years of education than participants with no to few depressive symptoms. A higher percentage of black participants reported more depressive symptoms than white participants. Also, women reported a higher percentage of depressive symptoms than men. Approximately 17% of participants reported loneliness, and 23% reported more depressive symptoms. About 54% of participants with more depressive symptoms also reported loneliness, compared to 5% of participants with no to few depressive symptoms. Participants who reported loneliness were also older and had fewer years of education than participants who were not lonely. A higher percentage of black participants than white participants were lonely, and more women were lonely compared to men.

**FIGURE 1.**
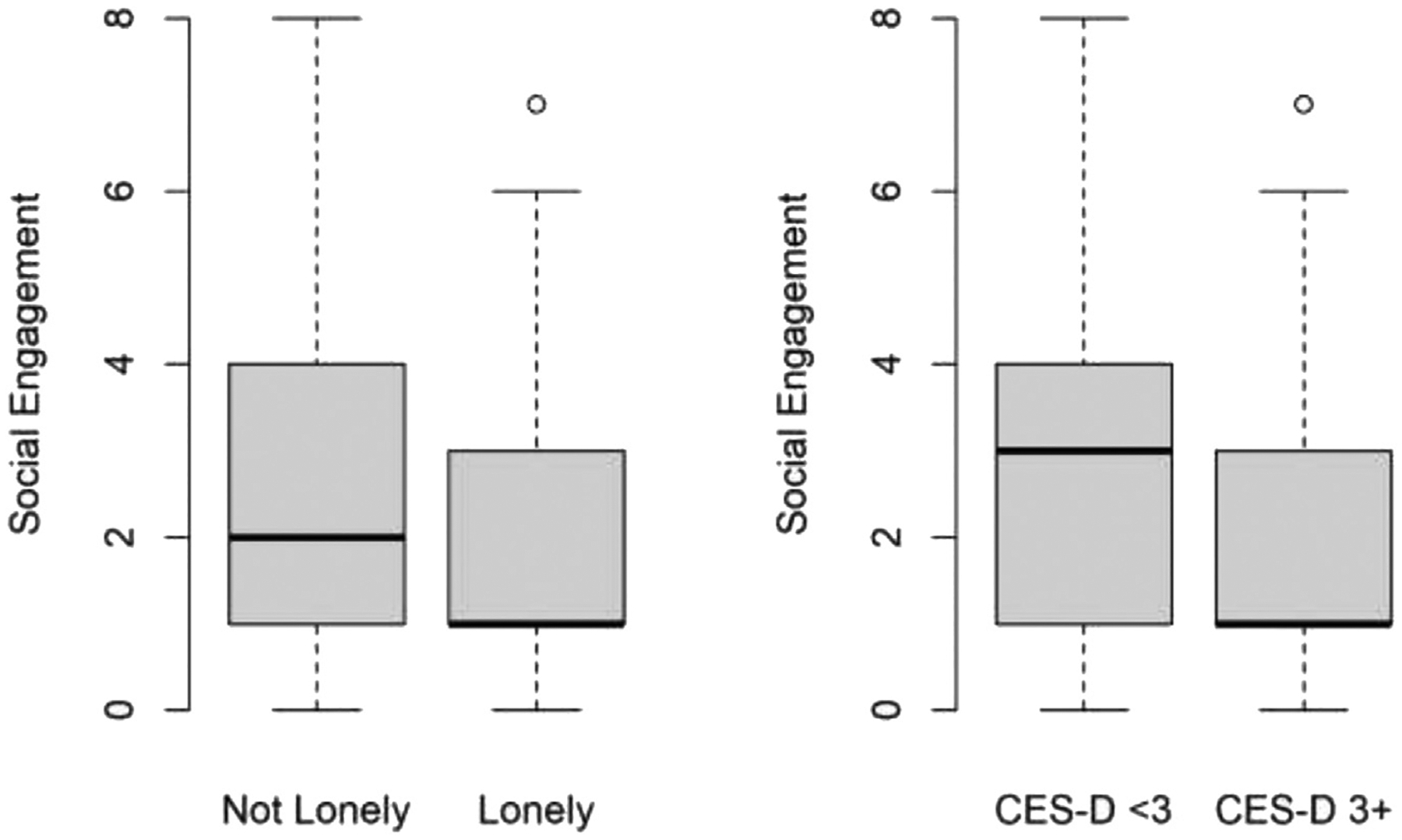
Social engagement in lonely vs. not lonely participants and in depressed vs. not depressed participants.

Figure 1 shows the distribution of social engagement at baseline among participants who were lonely and not lonely and among participants who were depressed and not depressed. The y-axis indicates social engagement score, and the x-axis represents either depressive status or loneliness status. We see a stark difference in the distribution of social engagement by loneliness status and by depressive status. Participants who were not lonely reported a had greater median social engagement at baseline than lonely participants. We observed a similar pattern for depressive status. Participants with no to few depressive symptoms had greater median social engagement at baseline than participants with more depressive symptoms.

The first column of Tables 2 and 3 provides the association between the frequency of social engagement and the annual rate of global cognitive decline for the total sample. Our findings show that social engagement was associated with a slower rate of annual cognitive decline (*β* = 0.007, SE = 0.001, Wald t-test = 7.00, df = 17,932, Wald t-test p-value <0.05). Thus, for each unit increase in social engagement, the rate of cognitive decline was slower by 0.007 Standard Deviation Units (SDU) per year or *β*, roughly a 13.5% (*β* = 0.007/*β* = −0.052) slower rate of cognitive decline for a unit increase in social engagement. This percent was calculated by dividing the *β* values for *Time × Social engagement/Time since baseline*.

Table 2 shows linear mixed effects regression analysis results, including a model with loneliness status as a predictor and stratified analysis by loneliness status. We did not find an interaction between social engagement, loneliness, and time on global cognition. However, we did find a statistically significant association between loneliness and baseline cognitive function (*β* = −0.099, SE = 0.017, Wald t-test = −5.82, df = 17,929, Wald t-test p-value <0.05). We conducted stratified analysis by loneliness status. In terms of loneliness, participants who were both lonely (*β* = 0.009, SE = 0.002, Wald t-test value = 4.50, df = 2,599, Wald t-test p-value <0.05) and not lonely (*β* = 0.006, SE = 0.001, Wald t-test value = 6.00, df = 15,326, Wald t-test p-value <0.05) experienced the benefits of social engagement on global cognition over time. Lonely individuals had a slower rate of cognitive decline of about 16.7% (*β* = 0.009/*β* = −0.054), and individuals who were not lonely had a slower rate of about 11.8% (*β* = 0.006/*β* = −0.051), for a one-unit increase in social engagement.

Table 3 shows linear mixed effects regression analysis results, including a model with depressive symptoms status as a predictor and stratified analysis by depressive symptoms status. We did not find an interaction between social engagement, depressive status, and time in global cognition. However, we did find a statistically significant association between the interaction of social engagement with depressive status on baseline global cognitive function. We conducted stratified analysis by depressive symptom status. Social engagement also decreased the rate of global cognitive decline in both participants who were depressed (*β* = 0.009, SE = 0.001, Wald t-test value = 9.00, df = 3,671, Wald t-test p-value <0.05) and those not depressed (*β* = 0.006, SE = 0.001, Wald t-test value = 6.00, df = 14,264, Wald t-test p-value <0.05). For an additional unit of increase in social engagement, participants with more depressive symptoms had a slower rate of cognitive decline at approximately 14.3% (*β* = 0.009/*β* = −0.063), as opposed to participants with no to few depressive symptoms who had a slower rate of decline at approximately 12.2% (*β* = 0.006/*β* = −0.049).

**FIGURE 2.**
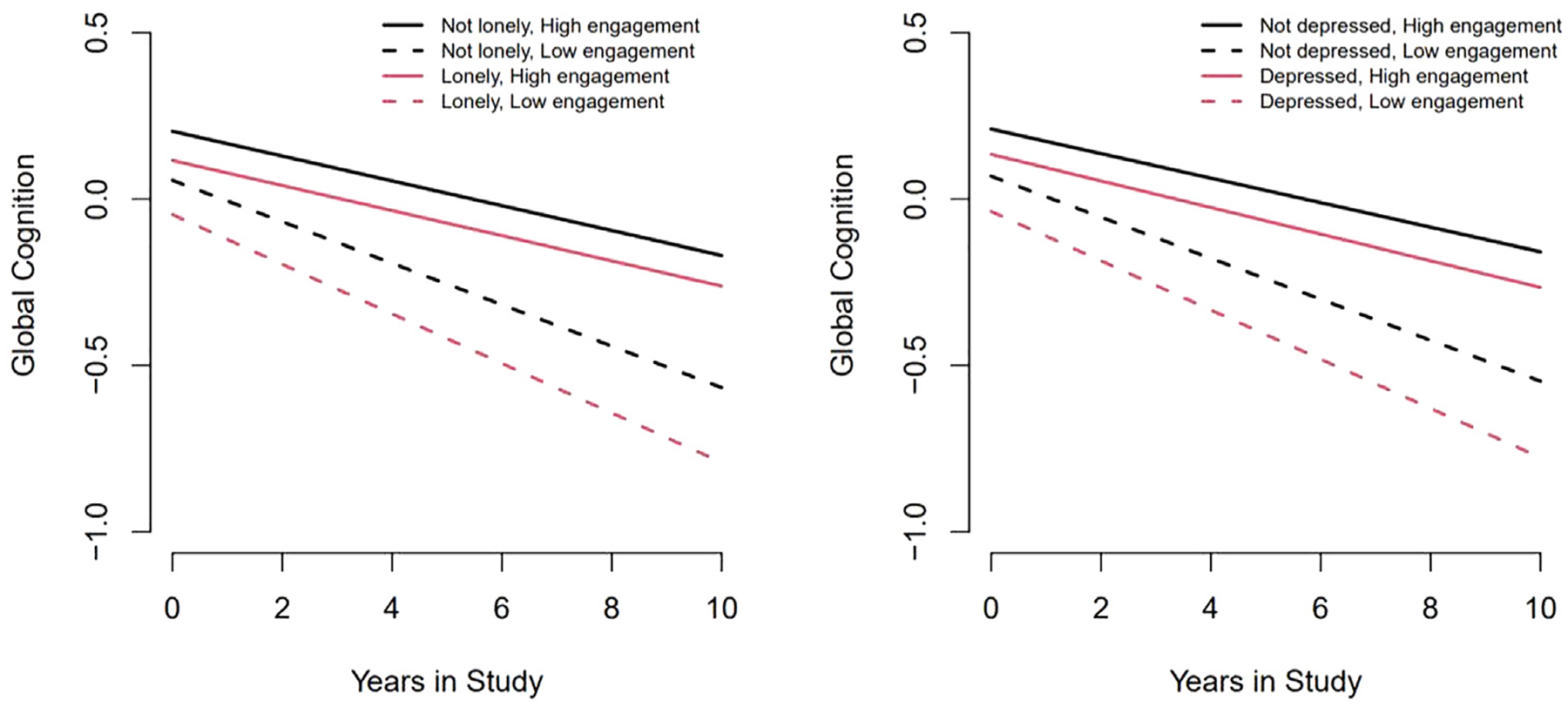
Ten-year cognitive decline in participants at the 10th and 90th percentiles of social engagement by loneliness and depression status.

Figure 2 shows that individuals with high levels of social engagement who are not lonely initially have better global cognitive function and slower global cognitive decline over time, followed by individuals with high levels of social engagement who are lonely, individuals with low levels of social engagement who are not lonely, and individuals with low levels of social engagement who are lonely. Individuals with high levels of social engagement who are not depressed initially have better global cognitive function and slower global cognitive decline over time, followed by individuals with high levels of social engagement who are depressed, individuals with low levels of social engagement who are not depressed, and individuals with low levels of social engagement who are depressed.

## DISCUSSION

Study results indicate that the interaction between loneliness and social engagement and the interaction between depressive symptoms and social engagement were both not statistically significant in their associations with cognitive decline, indicating that neither loneliness and depressive symptoms differentially influence the relationship between social engagement and cognitive decline. However, we still conducted stratified analysis to better understand within group associations among individuals experiencing more loneliness or more depressive symptoms. Older adults experiencing these feelings may be in greater need of tailored interventions specifically addressing the comorbidities of loneliness, depressive symptoms, and/or cognitive difficulties. Among lonely and nonlonely participants, we found statistically significant associations between social engagement and cognitive decline. Also, in the grouping of participants experiencing no to few depressive symptoms and in the grouping of participants experiencing more depressive symptoms, we also found statistically significant associations between social engagement and cognitive decline. These results indicate that social engagement may benefit cognition even in individuals experiencing more loneliness or depressive symptoms.

Social activities may be directly related to slowing cognitive decline or may be related to physical activities or mental health characteristics, influencing cognition.^38,39^ There are several theories which may explain underlying mechanisms between social engagement and cognitive decline, such as *cognitive reserve, stress*, and *disuse syndrome*.^40^ These theories indicate that taking part in activities which are interactive and enjoyable with mental engagement, such as regularly going to museums, can guard against dementia.^40^ Characteristics of the benefits of social engagement on cognition may depend on the population of focus.^41^ Among residents in long-term care, talking to and helping individuals may preserve cognition over time.^41^ Further, experiencing stress over time may have a negative impact on cognition, especially in women.^42,43^ Social engagement over the life course seems to impact the risk of dementia via cognitive reserve, decreasing stress with cerebrovascular benefits.^44^ Generally, participation in social activities in both middle and old age may reduce the risk of dementia from 30 to 50 percent.^44^ Our study helps to increase understanding of the associations between social engagement and cognitive decline in more vulnerable populations, such as individuals experiencing loneliness and/or depressive symptomology. We suggest that social engagement is protective of cognitive functioning over time, even in individuals who experience feelings of loneliness or depression. Our previous work showed that social engagement was associated with slowing rate of cognitive decline in CHAP participants.^45^ These findings support the need for this study as a next step, which showed that social engagement can benefit cognition even in individuals who experience loneliness and/or depression. The clinical implications of these results point to the importance of routinely screening for depression and/or loneliness and promoting opportunities for social engagement in older adults. This study has several weaknesses. We are measuring the association between baseline social engagement and cognitive decline. Therefore, we are not measuring social engagement over multiple periods. Other social activities may not be captured in the social engagement measure used for this study. Loneliness is measured using only a single question. Participants may not feel comfortable disclosing or may not remember their participation in social activities and experience specific depressive mood and loneliness characteristics. We compared the 10,572 participants in this analysis to the 230 participants who were omitted because they were missing measurements, such as CES-D (*n* = 188) or cognition (*n* = 55). The results of the t-tests showed a statistically significant difference in social engagement between excluded participants and those included. Participants who were not included in this study were less likely to be socially engaged than participants who were included, which is a limitation of the study. However, the number of excluded participants is small relative to the total sample in this study. Participants were primarily excluded due to having missing CES-D measurements. Please note that any causal relationships are speculative. There are also strengths to this study. The CHAP study consists of a large sample of black and white participants. We also had the opportunity to examine global cognition with multiple measurements over time.

A recent trial of 186 participants found that increased frequency and quality of social engagement may both be important to reduce dementia risk.^46^ Future research could expand upon this trial by examining how specific social activities and varying doses of participating in these activities may influence global cognitive decline.^46^ Additional research is needed to understand the relationships between social engagement and domains of cognitive functioning. Further, it is critical to determine if there are differences by demographic characteristics, such as age, sex, race, or socioeconomic status, in how social engagement is related to cognitive decline among individuals with and without loneliness or depression. More research is needed to increase knowledge of the mechanisms of action in the relationship between social engagement and cognition.^47^ Social engagement may change neural pathways or tissue.^47^ It may also be possible that social engagement increases brain volume, as indicated by research which found that rats that were in a setting with other rats had greater brain volume than those that were not.^47,48^ Socialization may offer neurocognitive benefits.^47,49^ Developing and applying methods to promote participation and adherence to social activities may be a feasible way to reduce the risk of cognitive impairment.

## Figures and Tables

**TABLE 1.** Baseline Sample of Study Participants in Total and by Depression and Loneliness Status

Variable	Overall, *N* = 10,572^a^	No to Few Depressive Symptoms^c^ N = 8,091^a^	More Depressive Symptoms^d^ N = 2,481^a^	df	p-value^b^
Age	73.2 (7.0)	72.9 (6.8)	74.3 (7.6)	10,570	<0.001
Female	6,494 (61%)	4,777 (59%)	1,717 (69%)	1.0	<0.001
Black	6,661 (63%)	4,792 (59%)	1,869 (75%)	1.0	<0.001
Education	12.3 (3.5)	12.6 (3.5)	11.0 (3.3)	10,570	<0.001
Lonely	1,757 (17%)	427 (5.3%)	1,330 (54%)	1.0	<0.001
Social engagement	2.34 (1.68)	2.53 (1.69)	1.69 (1.50)	10,568	<0.001
Global cognition	0.19 (0.78)	0.28 (0.73)	−0.08 (0.86)	10,564	<0.001
Study follow-up	8.8 (5.1)	9.0 (5.1)	8.1 (4.9)	10,570	<0.001
Variable	Overall, *N* = 10,561^a^	Not lonely *N* = 8,804^a^	Lonely *N* = 1,757^a^	df	p-value^b^
Age	73.2 (7.0)	72.8 (6.8)	75.0 (7.7)	10,559	<0.001
Female	6,487 (61%)	5,244 (60%)	1,243 (71%)	1.0	<0.001
Black	6,653 (63%)	5,408 (61%)	1,245 (71%)	1.0	<0.001
Education	12.3 (3.5)	12.5 (3.5)	11.1 (3.5)	10,559	<0.001
Social engagement	2.34 (1.68)	2.44 (1.69)	1.81 (1.55)	10,557	<0.001
Global cognition	0.19 (0.78)	0.25 (0.75)	−0.09 (0.87)	10,553	<0.001
Study follow-up	5.8 (5.1)	5.9 (5.1)	5.2 (5.0)	10,559	<0.001

a Mean (SD); n (%).

b Student’s t-test; Pearson’s Chi-squared test.

c CES-D score of 0−2: No to few depressive symptoms.

d CES-D score of 3−10: More depressive symptoms.

**TABLE 2.** Linear Mixed Effects Regression Analysis Results: Social Engagement and Cognitive Decline, by Loneliness Status

	Model 1	Model 2	Model 3	Model 4
	All participants Coefficient (SE) *N* = 10,565	All participants Coefficient (SE) *N* = 10,565	Lonely Coefficient (SE) *N* = 1,757	Not lonely Coefficient (SE) *N* = 8,808
Time since baseline	−0.052 (0.002)^a^	−0.051 (0.002)^a^	−0.054 (0.006)^a^	−0.051 (0.002)^a^
Social engagement	0.038 (0.003)^a^	0.035 (0.003)^a^	0.041 (0.009)^a^	0.037 (0.003)^a^
Time × Social engagement	0.007 (0.001)^a^	0.006 (0.001)^a^	0.009 (0.002)^a^	0.006 (0.001)^a^
Lonely		−0.099 (0.017)^a^		
Time × Lonely		−0.006 (0.003)		
Social engagement × Lonely		0.014 (0.008)		
Social engagement × Lonely × Time		0.002 (0.002)		

a Wald t-test value = Coefficient/SE, Wald t-test p-value <0.05.

Degrees of freedom: Model 1 = 17,932, Model 2 = 17,929, Model 3 = 2,599, Model 4 = 15,326.

All models adjusted for demographic characteristics, including age, sex, race, education, time, and their interactions with time.

**TABLE 3.** Linear Mixed Effects Regression Analysis Results: Social Engagement and Cognitive Decline, by Depressive Symptoms Status

	Model 1	Model 2	Model 3	Model 4
	All Participants Coefficient (SE) *N* = 10,565	All Participants Coefficient (SE) *N* = 10,565	Depressed (CES-*D* < 3) Coefficient (SE) *N* = 2,481	Not Depressed (CES-*D* ≥ 3) Coefficient (SE) *N* = 8,091
Time since baseline	−0.052 (0.002)^a^	−0.051 (0.002)^a^	−0.063 (0.006)^a^	−0.049 (0.002)^a^
Social engagement	0.038 (0.003)^a^	0.033 (0.003)^a^	0.043 (0.008)^a^	0.036 (0.003)^a^
Time × Social engagement	0.007 (0.001)^a^	0.006 (0.001)^a^	0.009 (0.001)^a^	0.006 (0.001)^a^
Depression		−0.094 (0.015)^a^		
Time × Depression		−0.008 (0.003)^a^		
Social engagement × Depression		0.017 (0.007)^a^		
Social engagement × Depression × Time		0.001 (0.001)		

a Wald t-test value = Coefficient/SE, Wald t-test p-value <0.05.

Degrees of freedom: Model 1 = 17,942, Model 2 = 17,939, Model 3 = 3,671, Model 4 = 14,264.

All models adjusted for demographic characteristics, including age, sex, race, education, time, and their interactions with time.

## Data Availability

The data has not been previously presented orally or by poster at scientific meetings.

## Highlights

- What is the primary question addressed by this study?
  Do depressive symptoms or loneliness differentially influence the association between social engagement and cognitive decline? What is the association between social engagement and cognitive decline in individuals with and without depressive symptoms or loneliness?
- What is the main finding of this study?
  Social engagement can slow cognitive decline even in individuals experiencing loneliness and/or depressive symptoms.
- What is the meaning of the finding?
  Strategies to increase social engagement should be incorporated into interventions which focus on reducing risk of cognitive impairment.
